# Multi‐Family Therapy in East and Southeast Asia—A Systematic Review and Meta‐Synthesis

**DOI:** 10.1111/jmft.70166

**Published:** 2026-08-19

**Authors:** Ouyang Hanlai, Sufang Peng, Jue Chen, See Heng Yim

**Affiliations:** ^1^ Shanghai Mental Health Center Shanghai Jiao Tong University School of Medicine and School of Psychology Shanghai China; ^2^ Department of Psychology University of Hong Kong HKSAR China

**Keywords:** East and Southeast Asia, family therapy, multifamily therapy, multiple family therapy, systematic review, systemic therapy

## Abstract

This systematic review examined the characteristics, cultural adaptations, and effectiveness of multi‐family therapy in studies conducted in the Chinese Mainland, Hong Kong, and Singapore. Eighteen reports from 17 studies were included, covering quantitative, qualitative, and mixed‐methods designs. Quantitative findings showed that multi‐family therapy was more consistently associated with improvements in individual‐level outcomes (e.g., symptom severity, psychological distress, functioning), whereas effects on family‐system outcomes were more mixed. Thematic synthesis of qualitative and mixed‐methods studies identified four interrelated themes: multi‐family therapy creates a non‐problem‐saturated space for families to come together; engages them in fun activities; fosters inter‐ and intra‐family connection; and supports relational understanding. Overall, individual‐level benefits are more readily captured by quantitative measures, while relational and process‐level changes may be less well‐reflected. Future research should strengthen methodological rigor and extend multi‐family therapy to presenting problems, such as eating disorders, to better evaluate its family‐system mechanisms and effects

## 1 Introduction

Multiple‐family therapy, or multi‐family therapy, is a therapeutic approach applied to various family problems and individual mental health conditions. It was first used as a treatment approach for families affected by schizophrenia (Laqueur et al. 1969), and has since been adopted across a range of conditions, including ADHD (Ma et al. 2022), conduct problems (Pérez‐García et al. 2020), anorexia nervosa (AN, Yim and White 2024), and bulimia nervosa (BN, Stewart et al. 2021). In the multi‐family therapy model, multiple families come together to work with a clinical team through various therapeutic activities in different formats (big group activity, separated young person, parent and sibling groups) to promote learning and maximize family resources. The process of change in multi‐family therapy involves several hypothesized interrelated mechanisms that contribute to therapeutic outcomes. One important aspect is the group dynamic created by bringing together multiple families facing similar challenges. Participants often experience connection and identification with others in comparable situations, which helps reduce social isolation and fosters hope (Baudinet et al. 2026). The intensity of the treatment and the exchange of new knowledge within the group are also described as influential in the change process, described by one of the creators as the “hothouse” effect (Asen et al. 2024).

Several systematic reviews have been conducted on understanding the impact of multi‐family therapy. A systematic review and meta‐analysis of 15 studies from North America and Europe on multi‐family therapy for eating disorders across the lifespan found significant benefits in weight gain, reduction of eating disorder symptoms, patient and parental distress symptoms, as well as reductions in parents' negative caregiving experiences (Zinser et al. 2022). However, these improvements were significant only when comparing pre‐ and post‐intervention measures and dissipated when multi‐family therapy was compared to other interventions. Family functioning, positive aspects of caregiving, and parental anxiety did not show improvement (Zinser et al. 2022). In a broader systematic review and meta‐analysis of multi‐family therapy across mental health conditions, 31 controlled studies were included, the majority of which were conducted in the United States and Western Europe (van Es et al. 2023). They found that due to high level of heterogeneity, it was difficult to draw conclusions regarding the effectiveness of multi‐family therapy. They found that multi‐family therapy was associated with improvements in schizophrenia symptoms, but little evidence was found for mood and conduct problems, and was associated with small improvements in family functioning. No study on eating disorders was included as the studies were not controlled, or included measures on family functioning. The authors argued that the added value of multi‐family therapy as an adjunct was not well defined‐future research needs to examine the potential working mechanisms of multi‐family therapy.

Family interventions are contextually situated, and culture plays an important role. Many of the multi‐family therapy interventions were conducted in Europe and the United States (van Es et al. 2023), which may not be directly applicable in Asian cultures. There are cultural specific factors in East and Southeast Asian cultures where collectivistic and individualistic values co‐exist, where families prioritize family harmony and face‐saving (face, i.e., social reputation and the avoidance of embarrassment or loss of respect), which could influence the help‐seeking behavior and family interactions in psychological interventions (Yim and Schmidt 2024). For example, a Singaporean study noted that “harmony” is both a central expectation that motivates Chinese families to seek therapy and a process variable that can constrain therapeutic progress by discouraging open discussion of conflict and differences. Accordingly, therapists need to maintain sufficient harmony to ensure safety and engagement, while introducing “manageable discord” at appropriate moments to facilitate change (Yim and Schmidt 2024). Literature on family therapy across China, Japan, South Korea suggests that harmony‐related norms may discourage overt conflict and limit open discussion of family difficulties and negative emotions (Tseng et al. 2020). Expectations of the therapist role are also culturally specific; in Hong Kong Chinese families, therapists may be viewed more as authoritative experts and providers of psychoeducation (Ma 2000). In addition, therapists need to acknowledge and respect key authority figures who often hold decision‐making power; if therapists challenge family authority figures too early—before a strong therapeutic alliance is established—it may lead to “loss of face,” thereby undermining engagement in multi‐family therapy (Sim et al. 2017). Participants often assumed that the therapists would take an active and expert role rather than a facilitator role (Loh et al. 2021; Kuge et al. 2024; Ma et al. 2017).

In a Hong Kong Chinese context, Ma et al. (2017) noted that parents of children with ADHD are expected to take active roles in educating their children, and often feel blamed and ashamed for the children's perceived misbehavior. Many are reluctant to disclose sensitive family issues to others, including professionals; families are hesitant to “wash dirty linen in public” (Loh et al. 2021). Although culturally collectivistic, addressing individual problems through a family lens is not seen as intuitive (Lee and Mock 2005; Loh et al. 2021). In a Hong Kong study, mindfulness exercise was seen as culturally relatable due to the influence from Buddhism and Daoism (Ma et al. 2017). Other structural adaptations include scheduling sessions on weekends as opposed to during consecutive days during the week to accommodate for working parents, especially fathers involvement, as fathers are often the assumed primary breadwinner (Lai et al. 2021). The traditional role where the mother is the homemaker is also strong in Japan (Kuge et al. 2024).

To our knowledge, there is no review that synthesizes the use of multi‐family therapy in East and Southeast Asia. The current systematic review and meta‐synthesis aimed to summarize and synthesize the use and effects of multi‐family therapy in East and Southeast Asia, as well as understand family's experiences of receiving multi‐family therapy, with a particular focus on understanding if specific cultural aspects influence the effects of multi‐family therapy. Different from a previous review (van Es et al. 2023), the study aimed to include non‐controlled studies, qualitative studies/mixed‐method studies, as well as controlled studies to ensure a broad understanding. Specifically, our review questions include the following:

What were the characteristics of the multi‐family therapy in East and Southeast Asia, including the presenting conditions, settings, and cultural adaptations?

What were the mental health and family functioning outcomes of these interventions?

What were the experiences of individuals and families who participated in multi‐family therapy?

## 2 Method

A review protocol was developed by the research team prior to commencing the review and is available in the Supplementary Information. Following the PICO (Population, Intervention, Comparison, Outcome) tool, the review included multi‐family therapy as an intervention for any mental health disorder. Comparison groups included care as usual, control or uncontrolled designs, measuring the outcomes of mental health symptoms and/or family relationships.

A database search was first conducted in April 2025. Four databases were searched (APA PsycInfo, Ovid Medline, Global Health, and Embase), and a Chinese database (China National Knowledge Infrastructure, CNKI) was searched for articles available only in Chinese. Search terms included variations of multi‐family therapy and regions in East and Southeast Asia for each database (see Supporting Information). Handsearching was then carried out by (a) reviewing the reference lists of included studies and relevant systematic reviews, and (b) consulting with experts in multi‐family therapy (Ma, J., personal communication, September 12, 2025). This process identified three additional records, which were added to the database search results before duplicate removal.

All identified records (184 from database searches and 3 from handsearch) were imported into EndNote. Duplicates were removed using EndNote's automatic duplicate detection feature, followed by manual verification based on author names, titles, and publication years. A total of 45 duplicates were removed, leaving 142 records for title and abstract screening. The expert consultation served to identify any missing published or unpublished studies known to field experts that were not captured by the database searches.

### 2.1 Inclusion and Exclusion Criteria

Studies were included if they were peer‐reviewed articles with pre‐post outcome measures (including controlled, uncontrolled, and mixed‐method designs) or qualitative studies; focused on multi‐family therapy; were conducted in East and Southeast Asia as defined by Encyclopedia Britannica (https://www.britannica.com/); and were published in English or Chinese. Dissertations, conference abstracts, and book chapters were excluded. Additionally, descriptive case studies were excluded because they typically lack pretest measurements and therefore did not satisfy our requirement for pre‐post outcome measures or a clearly described analytic process. Article titles and abstracts were screened independently by two researchers (OYHL & SHY). Based on this screening, full‐text articles were retrieved and independently assessed. Inconsistencies were resolved following discussions between the research team. Three case studies were excluded. Two further articles were also excluded at full‐text screening: one protocol paper that only outlined a planned multi‐family therapy trial without reporting outcome data, and one article on ADHD and maltreatment (Ma et al. 2016) that did not present evaluative outcomes of a multifamily intervention.

### 2.2 Data Extraction

The following data were extracted: study type, condition treated, region, treatment setting, cultural elements, theoretical framework, and group components, outcome measure, and effectiveness. For qualitative studies or mixed‐method studies, themes and individual participants' quotes were extracted. Data extraction was conducted by one reviewer, O.Y.H.L., and independently checked by a second reviewer S.H.Y. Discrepancies were resolved through discussion.

### 2.3 Quality Appraisal and Analysis

Due to the heterogeneity of the studies, the mixed‐method appraisal tool (MMAT) (Hong et al. 2018) was used. To assess the methodological quality and risk of bias of the included studies, the first author appraised the studies using the Mixed Methods Appraisal Tool (MMAT) (Hong et al. 2018). The first author conducted the appraisal, and a 10% of the included studies were also rated by the corresponding author. The aim was to enrich the analysis rather than establish reliability. For each paper, we first applied the two MMAT screening questions to confirm its eligibility as an empirical study, and then selected the appropriate study category (qualitative, quantitative randomized, quantitative non‐randomized, quantitative descriptive, or mixed methods) based on the reported methods. Within the relevant category, we rated the five methodological criteria as “Yes,” “No,” or “Can't tell,” with the latter indicating insufficient or unclear reporting. The authors of the tool did not advocate for scoring the studies, but instead, the appraisal complemented the interpretation of the findings. Studies would not be excluded based on the appraisal.

Due to substantial clinical and methodological heterogeneity across the included studies, a meta‐analysis was not conducted. Specifically, the studies varied considerably in terms of clinical populations (e.g., ADHD, depression, psychosis), intervention formats (e.g., duration, session frequency, number of sessions), cultural adaptations, and outcome measures (e.g., different validated scales assessing distinct constructs). Given that pooling effect sizes under such heterogeneity would yield statistically questionable and clinically uninterpretable results, a narrative synthesis (Popay et al. 2006) was adopted for the quantitative and mixed‐method studies. This process included tabulation and grouping, assessing the strength of the evidence, and identifying main findings.

For the qualitative studies and qualitative aspects of mixed‐method studies, thematic synthesis method was chosen for its suitability in understanding people's views and experiences of multi‐family therapy to inform clinical practice (Thomas and Harden 2008). Different from meta‐analysis, qualitative meta‐synthesis places emphasis on interpretative explanations. The corresponding author, S.H.Y., conducted the synthesis and followed multiple steps, first extracting the texts (participants' quotes, themes, and subthemes of the included studies) and coding them line‐by‐line. The coding and theme development process was primarily inductive, allowing the generation of themes in a bottom‐up manner rather than being imposed by a predetermined framework. Preliminary codes and themes were reviewed in regular team meetings, where alternative interpretations and ambiguities were discussed and refined through consensus among the authors. Where necessary, a third team member was consulted to support the interpretation of the findings. Similar codes were then grouped and organized into descriptive themes. The next stage involved generating analytical themes from the descriptive themes, enabling the development of new interpretations. In line with established methods of qualitative synthesis, the quotations presented in the results section were drawn directly from the original studies included in this review and selected based on their relevance to the identified themes to illustrate the synthesized findings (Barnett‐Page and Thomas 2009). These stages were iterative rather than linear, and regular discussions were held among the research team throughout the process. This approach allows researchers to synthesize qualitative research findings systematically while maintaining a transparent connection to the original data.

### 2.4 Declaration of Use of Artificial Intelligence Tools

ChatGPT was used for refining Grammar and language, as the authors are not native English speakers.

### 2.5 Reflexivity

All authors have experience delivering multi‐family therapy in the context of eating disorders. SHY is a clinical psychologist and researcher, OYHL is a postgraduate student and psychotherapist in training, and SFP and JC are consultant psychiatrists. All authors have attended a 3‐day intensive training workshop on multi‐family therapy in Asia context.

The authors' clinical experience in delivering multi‐family therapy for eating disorders informed the design and conduct of this review, particularly in shaping the research questions and interpreting the findings. Although no studies on eating disorders met the inclusion criteria, this familiarity provided a lens for understanding how multi‐family therapy is implemented across different conditions. The authors acknowledge that prior clinical experience may have led to certain expectations regarding the effectiveness of multi‐family therapy; nevertheless, this review adhered to a systematic methodology with predefined inclusion criteria to minimize bias.

In addition, the authors are situated within cultural contexts where family harmony and relational interdependence are highly valued. This may have influenced the interpretation of themes such as togetherness, communication, and relational understanding.

## 3 Results

**Figure 1:**
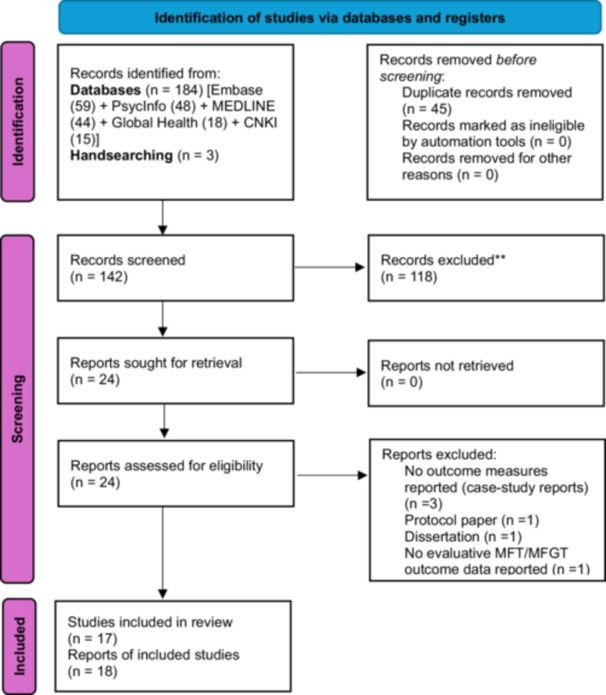
PRISMA flow diagram. [Color figure can be viewed at wileyonlinelibrary.com]

A total of 187 records were identified, comprising 184 records from database searching (Embase, APA PsycInfo, Ovid MEDLINE, Global Health, and CNKI) and 3 records from handsearching. After removal of 45 duplicate records, 142 records remained for title and abstract screening, of which 118 were excluded as not meeting the inclusion criteria. The full texts of 24 reports were retrieved and assessed for eligibility. Six reports were excluded at this stage: 3 case‐study reports without outcome measures, 1 protocol paper, 1 dissertation, and 1 study reporting no evaluative multi‐family therapy outcome data, leaving 18 reports from 17 studies to be included. The study selection process is summarized in the PRISMA flow diagram (Figure 1).

### 3.1 Results of Quality Appraisal

Using the MMAT, criterion‐level ratings for each study are presented in Supporting Information S1: Tables S1 and S2 to provide a detailed picture of study quality. The quality appraisal was used to describe the methodological strengths and limitations of the included studies and to contextualize the findings. No studies were excluded based on quality scores, and the appraisal did not assign differential weight to studies in the narrative synthesis, consistent with the aim of providing a comprehensive overview of available evidence.

Across the included studies, overall methodological quality was generally moderate, with clearer strengths in measurement appropriateness and intervention delivery. The RCTs met most MMAT criteria; however, several key methodological details (e.g., randomization procedures, baseline comparability, and assessor blinding) were rated as “Can't tell,” reflecting insufficient reporting and reducing certainty about risk of bias. Quality concerns were more prominent in non‐randomized quantitative studies, where representativeness of participants (MMAT 3.1) was frequently rated “No/Can't tell,” indicating potential selection bias and limited generalizability. Incomplete outcome data (MMAT 3.3) and attrition were also common, particularly in pre–post designs, which may bias effect estimates. Although confounding was addressed in some studies (MMAT 3.4), baseline imbalances were not consistently controlled. Mixed‐methods studies generally demonstrated a sound rationale and integration, yet some provided limited handling of divergences between qualitative and quantitative findings. Qualitative studies were largely robust, although several were rated “Can't tell” for the transparency of how findings and interpretations were derived from data.

### 3.2 Characteristics of the Studies

Table 1 summarizes key characteristics of the included studies, such as population and setting, target disorder, cultural components, theoretical framework, and multi‐family therapy group design. Across the 18 included articles published between 2004 and 2025, there were nine quantitative studies (two of which were RCTs), three mixed‐methods studies, and six qualitative studies. The included studies were conducted in the Chinese Mainland (*n* = 4), Hong Kong (*n* = 12), and Singapore (*n* = 2).

**Table 1:** Summary of intervention characteristics.

Type of study	Author (year)	Disorder	Region	Setting (inpatient/outpatient; adult/children)	Cultural sensitivity/component/adaptation	Theoretical framework	Design of the MFT group
*RCTs*							
Quantitative (RCT)	Chien and Chan (2004)	Schizophrenia	Hong Kong	Outpatient/96 adult patients and 96 primary family caregivers; 65 families in MFT group (caregivers only)	Using culture‐specific Chinese family structures (extended family, interdependence, filial responsibility, mutual support) within group interventions	Family systems theory	Intervention: 12 biweekly, peer‐led group sessions (2 h/session). Control: 12 biweekly, clinician‐led psychoeducation sessions (2 h/session); standard care group.
Quantitative (RCT)	Zhuo et al. (2020)	Schizophrenia (stable phase)	Chinese Mainland	Inpatient transitioning to outpatient/60 schizophrenia (aged 18–65) and their primary caregivers; 30 families in MFT group	Family‐centered intervention with Chinese kinship dynamics	Biopsychosocial rehabilitation model	Intervention: Two‐phase program—Intensive (inpatient): 10 sessions, twice weekly (90 min/session), four to six families per group; Consolidation (outpatient): two monthly sessions (90 min/session). Control: Routine outpatient care.
*Non‐randomized controlled studies*							
Quantitative (Nonrandomized)	Lai et al. (2021)	ADHD	Hong Kong	Outpatient/81 families caring for ADHD children (aged 6–12) and at least one parent per family; 39 families in MFT group	Addressing Hong Kong Chinese sociocultural context (emphasis on academic success, obedience, and parental role differences)	Adapted from the MFT model developed in the UK by (Asen and Scholz 2010), details reported in Ma et al. (2011)	Intervention: Six‐month, weekend‐based program (42 h in total)—one psychoeducational talk (4 h), four full‐day sessions (32 h in total), and two half‐day reunions (6 h in total); four to seven families per group. Control: two half‐days of psychoeducational talks over a 3‐month period
Quantitative (Nonrandomized)	Liu et al. (2015)	Internet addiction	Chinese Mainland	Outpatient/46 adolescents and their parents; 21 families in MFT group	Not reported	Family systems theory	Three‐week, every‐3‐days program (12 h in total)—six 2‐h sessions (12 h); seven families per group (three groups; 21 families).
Quantitative (Nonrandomized)	Ma et al. (2018)	ADHD	Hong Kong	Outpatient/114 parents from 82 families of children with ADHD; 61 parents/40 families in MFT group	Mindfulness exercises were framed as consonant with Chinese Buddhist, Confucian, and Daoist traditions and adapted to crowded living conditions	MFT model (Asen and Scholz 2010), details in Ma et al. (2017)	Six‐month, weekend‐based program (42 h in total)—one psychoeducational talk (4 h), four full‐day sessions (32 h in total), and two half‐day reunions (6 h in total); four to seven families per group.
Quantitative (Nonrandomized)	Ma et al. (2022)	Depression	Hong Kong	Outpatient/76 children of parents with depression; 51 families in MFT group	Focus on the effects of depression on individual and family well‐being	MFT model (Asen and Scholz 2010), details in Ma et al. (2018)	Same as Ma et al. (2018)
Quantitative (Nonrandomized)	Ma et al. (2023)	Depression	Hong Kong	Outpatient/61 parents with depression(parents of the above child sample); parents only	Focus on the effects of the disorder on family life, rather than on the pathology of the depressed parent/s	MFT model (Asen and Scholz 2010), details in Ma et al. (2018)	Same as Ma et al. (2018)
Quantitative (Nonrandomized)	Wang et al. (2016)	Childhood schizophrenia remission phase	Chinese Mainland	Inpatient + outpatient/113 Children in remission and their parents; 45 families in MFT group	Not reported	Family systems theory	Weekly sessions (90 min/session) for 16 sessions; four families per group.
Quantitative (Nonrandomized)	Zhang et al. (2022)	Bipolar disorder (BD) remission phase	Chinese Mainland	Inpatient/83 Adult BD patients and at least one parent per family; 41 families in MFT group	Not reported	Biopsychosocial model + Family systems theory	Two‐phase program—Intensive (inpatient): 10 sessions, twice weekly (90 min/session), four to six families per group; Consolidation (outpatient): two monthly sessions (90 min/session).
Mixed‐methods (Non‐randomized)	Ma et al. (2019)	ADHD	Hong Kong	Outpatient/88 ADHD children (aged 5–12) and their parents; 43 families in MFT group	Respond to the needs of Chinese families of children with ADHD; Families are competent and resourceful	MFT model (Asen and Scholz 2010), details in Ma et al. (2018)	Three‐month, mostly weekend‐based program (40 h in total)—one psychoeducational talk, four full‐day group activities, and one half‐day reunion; four to eight families per group.
*Uncontrolled feasibility studies*							
Mixed‐methods (uncontrolled feasibility studies)	Lo et al. (2024)	Autism Spectrum Disorder	Hong Kong	Outpatient/13 Adult ASD patients and at least one parent per family	Cultural framing of Chinese families' values (familial obligation and stigma)	MFT model (Ma et al. 2018)	Three‐month, mostly weekend‐based program (38 h in total)—one psychoeducational talk, four full‐day group activities, and one half‐day reunion; four to eight families per group.
Mixed‐methods (uncontrolled feasibility studies)	Loh et al. (2023)	First‐Episode Psychosis (FEP)	Singapore	Outpatient/17 patients and their 38 family members	Recognizes the limits of Western psychoeducational models in East Asian contexts to better fit Singaporean families' needs.	MFT model (Asen and Scholz 2010), Narrative therapy techniques	Weekly, four sessions (3 h/session) delivered over 4 weeks.
*Qualitative studies*							
Qualitative study	Lo and Ma (2023)	ADHD	Hong Kong	Outpatient/13 patients (aged 11–15) and 14 parents	Grounded in the Hong Kong Chinese context (stigma, limited social support, high‐density urban environment); Incorporating local natural outdoor settings	MFT model (Asen and Scholz 2010), therapy in nature (Burg 1994)	Three‐month, mostly weekend‐based program (40 h in total)—two full‐day, campus‐based sessions, a two‐day/one‐night outdoor multi‐family camp, and one full‐day campus reunion.
Qualitative study	Lo and Ma (2024)	ADHD	Hong Kong	Outpatient/14 adolescents and 20 parents	Adapts Ma et al.'s (2017) MFT model into the MFT‐FDV format, adding a wilderness family camp with a strong mutual‐support focus for Chinese families	Family systems theory, developmental theory, and group theory	Same as Lo and Ma (2023)
Qualitative study	Loh et al. (2021)	First episode psychosis	Singapore	Outpatient/9 Adult FEP(aged 16‐40) and 10 caregivers	Start with practical issues, deepening sharing as safety builds (low initial self‐disclosure); use an active, structured, expert‐led style to build trust (expectations of professional authority)	MFT model (Asen and Scholz 2010)	Four weekly sessions and a follow‐up session
Qualitative study	Ma et al. (2011)	Child behavioral/emotional issues:	Hong Kong	Outpatient/14 children (aged 5–12) from 11 families and 11 family members	With pre‐group psychoeducation to meet Chinese parents' treatment expectations, and activities structured around respect for familial hierarchy and boundaries	MFT model (Asen and Scholz 2010)	Four half‐day sessions; one evening psychoeducational talk (2 h) plus three days of joint family activities, including one parallel parent/child session
Qualitative study	Wan et al. (2016)	ADHD	Hong Kong	Outpatient/21 ADHD Children from 20 families and 34 family members	Culturally informed rationale (face, stigma, insider–outsider norms)	MFT model (Asen and Scholz 2010); “co‐researching” framework (Epston 1999)	Four parent‐child conference lasts for an hour
Qualitative study	Wong et al. (2019)	Parent–child relationship problems	Hong Kong	Inpatient and outpatient/53 Children from 32 families and 38 family members	MFT model for Hong Kong Chinese families (respecting hierarchy/boundary, pre‐group psychoeducation)	Attachment theory; Social support theory	Weekend‐based program—one psychoeducation talk (2 h), four intensive group days across four consecutive weekends, and post‐group reunion sessions; three to six families per group

### 3.3 Participant Characteristics

Participants were recruited from both child and adult mental health services, with 11 studies focusing on children and adolescents and seven studies focusing on adult populations. Fourteen of the 18 studies were conducted in outpatient or community settings, whereas four studies involved inpatient settings. Among the quantitative studies, four relied primarily on parent‐reported outcomes, six assessed patient outcomes only, and two included both parent‐ and patient‐reported measures.

### 3.4 Multi‐Family Therapy Design

Three broad categories of multi‐family therapy design were identified. The first consisted of intensive, short‐term multiple‐family programmes primarily developed in Hong Kong (Lo et al. 2024; Lo and Ma 2023, 2024; Ma et al. 2018, 2019, 2022, 2023), characterized by an initial psychoeducational session followed by concentrated multi‐family activities and consolidation meetings, typically delivered in small groups of families. The second category included briefer formats delivered weekly, biweekly (Chien and Chan 2004; Wang et al. 2016), or in short intensive cycles (Liu et al. 2015), ranging from four‐session models to time‐limited group programmes. The third category comprised interventions spanning both inpatient and outpatient phases, combining intensive inpatient group work with subsequent outpatient consolidation (Zhuo et al. 2020; Zhang et al. 2022).

### 3.5 Outcome Measures Used

The following outcome measures were identified across the included studies. A summary of all the names of the scales used in the study, along with detailed references, is provided in Supporting Information S1: Table S3.

#### 3.5.1 Symptom and Functioning Outcomes

Symptom severity and individual functioning were commonly assessed using standardized clinician‐rated, parent‐rated, or self‐report measures, with substantial variation across diagnostic groups (see Supporting Information S1: Table S4). For example, studies involving schizophrenia and bipolar disorder frequently used symptom and functioning scales such as Positive and Negative Syndrome Scale (PANSS) and Personal and Social Performance Scale (PSP), whereas ADHD studies primarily relied on parent‐rated measures, specifically the Strengths and Weaknesses of ADHD Symptoms and Normal Behavior Scale (SWAN).

#### 3.5.2 Family‐Level and Parent‐Related Outcomes

Family‐ and parent‐related outcomes were commonly assessed using self‐report measures focusing on family functioning, parent‐child relationships, parenting stress, and parenting self‐efficacy (see Supporting Information S1: Table S4). Frequently used instruments included measures of family functioning and parent–child interaction (e.g., General Family Functioning Scale [GFFS], Parent–Child Relationship Scale [PCR], Parent–Child Communication Scale [PCCS]), as well as parent‐reported indicators of psychological distress and caregiving resources (e.g., Parenting Stress Index [PSI], Parenting Sense of Competence Scale [PSOC]).

### 3.6 Effectiveness

#### 3.6.1 Evidence From Randomized Controlled Trials (RCTs)

Two RCTs evaluated multi‐family therapy for schizophrenia and consistently reported superior outcomes relative to control conditions. Participants receiving multi‐family therapy demonstrated greater improvements in social functioning (Personal and Social Performance Scale [PSP]), depressive and anxiety symptoms (Self‐Rating Depression Scale [SDS]/Self‐Rating Anxiety Scale [SAS]), and life satisfaction (Life Satisfaction Index A [LSIA]), with some effects maintained at 3‐month follow‐up (Chien and Chan 2004; Zhuo et al. 2020). Neither RCT included family‐system outcomes as outcome variables.

#### 3.6.2 Evidence From Non‐Randomized Controlled Studies

Eight non‐randomized controlled studies examined multi‐family therapy across childhood schizophrenia, bipolar disorder, ADHD, adolescent internet addiction, and parental depression. Overall, findings suggested benefits for symptoms and individual functioning. Compared with control conditions, multi‐family therapy was associated with greater reductions in psychiatric symptoms (Positive and Negative Syndrome Scale [PANSS]) and improvements in social functioning (Personal and Social Performance Scale [PSP]) among children with schizophrenia (Wang et al. 2016), higher medication adherence and improved social functioning among individuals with bipolar disorder (Zhang et al. 2022), and greater reductions in internet addiction severity, with effects maintained at follow‐up (Liu et al. 2015). In ADHD programmes, studies reported greater reductions in parent‐rated ADHD symptom severity (e.g., Strengths and Weaknesses of ADHD Symptoms and Normal Behaviour Scale [SWAN]; effect sizes 0.56–0.69; Lai et al. 2021; Ma et al. 2018). Findings for family‐level and parental outcomes were less consistent. Across ADHD studies conducted in Hong Kong, no significant between‐group differences were observed for parental stress, parental self‐efficacy, or parent–child relationship quality (Lai et al. 2021; Ma et al. 2018, 2019). One exception was a small but sustained reduction in parental psychological distress (Brief Symptom Inventory [BSI]) among parents with depression receiving multi‐family therapy (Ma et al. 2023). In addition, one study targeting adolescent internet addiction reported significant improvements in parent–child closeness and communication, with effects maintained at 3‐month follow‐up (Liu et al. 2015).

#### 3.6.3 Evidence From Uncontrolled Studies

Two uncontrolled feasibility studies evaluated multi‐family therapy programmes for high‐functioning autism spectrum disorder (HF‐ASD) and first‐episode psychosis (FEP). In the HF‐ASD programme, online multi‐family therapy was associated with improved participant‐rated family relationships (*d* = 0.68; Lo et al. 2024). The FEP programme was evaluated primarily in terms of feasibility and acceptability rather than effectiveness, with high satisfaction ratings reported by clients and carers (Loh et al. 2023).

#### 3.6.4 Summary of Findings

Overall, evidence from RCTs and non‐randomized controlled studies suggests that multi‐family therapy is associated with improvements in symptoms and individual functioning across several clinical populations. In contrast, evidence for parenting, parent–child relationships, and broader family‐level outcomes remains mixed and less conclusive. Findings from uncontrolled feasibility studies provide preliminary support for acceptability and potential benefits but offer limited evidence regarding effectiveness.

### 3.7 Thematic Synthesis of Qualitative and Mixed‐Method Studies

Ten qualitative or mixed‐method studies explored experiences of multi‐family therapy. Most studies were conducted in Hong Kong and one study in Singaporean context. In terms of conditions, studies primarily focused on ADHD, autism, and psychosis. Supporting Information S1: Table S5 lists the studies' themes and subthemes.

**Figure 2:**
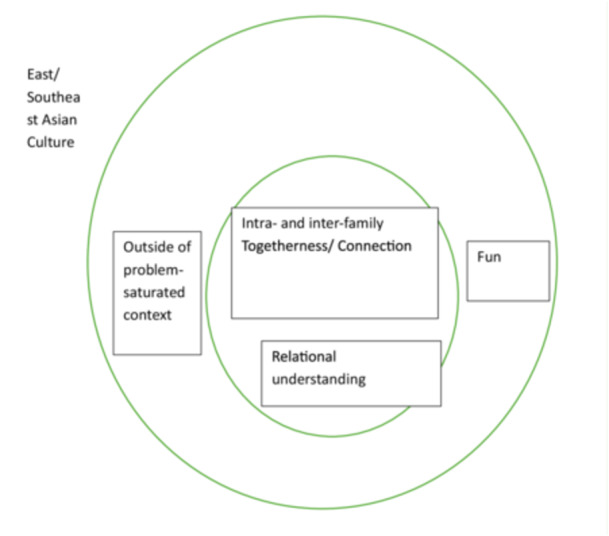
Thematic map. [Color figure can be viewed at wileyonlinelibrary.com]

Thematic synthesis of all the qualitative and mixed‐method studies yielded four themes presented in a thematic map (Figure 2) with different layers. multi‐family therapy provides a (1) non‐problem‐saturated context for families to come together, (2) engaging in fun activities, where the (3) inter‐ and intra‐family connection and togetherness provides a basis for (4) relational understanding to be fostered. All these findings are situated within the cultural contexts represented in this review, where the participants were predominantly ethnic Chinese, where family honor and the need to “save face” further worsens the stigmatization of mental health issues and family conflicts. By providing a psychologically safe setting, the need to “save face” is mitigated by shared experience across the families. As an illustrative example, a participant said, “I would not be afraid of being stigmatized in this group. We would therefore share openly—both the good and bad sides of our family” (Lo and Ma 2024). Another participant shared similar thought: “Nobody would look down on you because we have similar family backgrounds and relationship problems with our parents” (Wong et al. 2019).

#### 3.7.1 A Non‐Problem‐Saturated Context

Multi‐family therapy provides a safe and unique context where the families can articulate their feelings and view their children and problem from a fresh perspective. For example, one of the multi‐family therapy took place in nature where families had to set up tents and engage in camping (Lo and Ma 2024). Multi‐family therapy provided a crucial break from the day‐to‐day problem‐saturated routine. A parent said, “We would stay home for the whole day if we didn't join the group… Either I would be pushing him to study or he would lock himself in the room playing computer games… Coming here gave us a break” (Lo and Ma 2023). Many families shared that they were able to talk about issues and topics not talked about during normal interactions, for example, parents' own upbringing (Wong et al. 2019) and inner thoughts (Loh et al. 2021). One participant shared, “It's strange to talk about difficulties with social relationships at home… but I feel safe with saying so here” (Lo et al. 2024).

#### 3.7.2 Fun Activities

The inclusion of relaxing, enjoyable, and creative activities, especially those that utilized the outdoors for young people with ADHD, provided necessary relief from daily stressors and facilitated positive interaction. Children participants described the group as a “cosy and comfortable place” (Ma et al. 2019) and found most of the activities fun, interesting and creative. Outdoor activities were particularly positively received, which provided freedom and autonomy in an open area (Wong et al. 2019). In a photo‐elicitation study, one mother noted that camping was “like a family trip… nothing to worry about on the trip… this helped us relax together” (Lo and Ma 2023). Children cherished the positive family interaction during the fun family activities, such as one participant being impressed by their father's smile while kicking a ball with him after lunch, because “dad seldom smiles at home or plays ball with me” (Ma et al. 2019).

#### 3.7.3 Intra‐ and Inter‐Family Togetherness/Connection

A key theme across the studies was the connection both within and between the family units, offering peer support, friendships, solidarity, and practical insights among families facing similar challenges. Multi‐family therapy was seen as a supportive community space to share and for families to come together as they did not share the feelings and the thoughts to their friends and families (Lo and Ma 2024). The families no longer felt they were facing the conditions/problems alone: “Sharing of experience from other families make me realise that I am not the only one in this situation.” (Loh et al. 2021). In terms of within family units, participants used a lot of “we” language. Some of them used migratory birds as an analogy, “Migratory birds live as a flock. They do everything together, and they migrate together. Just like us in the group, we play together and we do other things together” (Ma et al. 2019). Participants noticed the family bond and strength beyond the difficulties the family faced: “Of course the difficulty is still there… but I realized that my family actually has strong cohesion… we would do things together and committedly” (Lo and Ma 2023).

#### 3.7.4 Relational Understanding

Multi‐family therapy facilitates a perceptual shift in parents, helping them move away from problem‐focused narratives towards recognizing unique strengths and values of their children. This increased understanding possibly facilitated an improved communication and family cohesion. For instance, a parent noted, “When we played freely on the grassland…I felt carefree and became more attentive to his strengths rather than being picky about his problems…this is a dramatic change for me” (Lo and Ma 2023). One mother realized her daughter had strengths she had never noticed at home and that she could now accept and tolerate her weakness more—“I scold her less now” (Ma et al. 2011). The understanding is relational and mutual, as the children also learnt about their parents' upbringing and got an insight into their ways to expressing care (Wong et al. 2019). Children reported that parents listened to them more, and understood them more (Ma et al. 2019).

In East/Southeast Asian culture, parenting beliefs are shaped by interdependence (Chao and Tseng 2002). Through taking part in multi‐family therapy, parents began to focus on fostering independence of their children. Noticing the child's strengths that she did not realise before, a parent shared, “he's (her son) mature and independent and has his own ideas… I shouldn't treat him like a baby anymore” (Lo et al. 2024). Multi‐family therapy provided a context for observational learning where some participants termed it “mirroring”: “The group is like a mirror. I can understand myself more by looking at others who are similar to me. I can also observe how other group members get along with their parents. I've learned from that” (Lo et al. 2024).

## 4 Discussion

This review synthesized evidence on the characteristics, cultural adaptations, and effectiveness of multi‐family therapy in studies conducted in the Chinese Mainland, Hong Kong, and Singapore, integrating both quantitative and qualitative findings. Overall, quantitative findings showed that multi‐family therapy was more consistently associated with improvements in individual‐level outcomes, whereas findings relating to relational and family‐level outcomes were less consistent based on the current state of evidence.

### 4.1 Evidence for Individual‐Level Outcomes

To our knowledge, this is the first systematic review to specifically examine the intervention effects of multi‐family therapy in East and Southeast Asia, drawing on studies conducted in the Chinese Mainland, Hong Kong, and Singapore. Overall, the evidence was stronger for individual‐level outcomes (e.g., symptom severity, psychological distress, and functioning) than for family‐system outcomes (e.g., parent–child relationships, family functioning, and parenting resources). This pattern is consistent with previous reviews, which have reported more robust effects of multi‐family therapy on symptom‐related outcomes than on broader family functioning across diagnostic groups (Baudinet et al. 2021; van Es et al. 2023).

One possible explanation for this pattern relates to the focus of the interventions. Most interventions included transdiagnostic relational components such as multi‐family tasks, experiential activities, and facilitated sharing. However, these components were largely designed to support illness management and recovery, rather than to directly target broader family‐system functioning. As a result, many activities that appeared “relational” in form were explicitly anchored to the illness context. For instance, the “Flying Eggs” exercise developed by Ma and colleagues required parents to follow their child's instructions, helping parents recognize anxiety‐driven parenting behaviors when caring for adolescents with ADHD (Ma et al. 2017). Similarly, the “Family Tree” activity described by Loh et al. (2023), though framed as an exploration of family relationships, primarily targeted illness‐related historical coping patterns to support caregiving. In this way, such activities were more likely to modify illness‐related family interactions (e.g., caregiving consistency, reduced blame and conflict, and increased understanding and support), which may translate more readily into individual‐level improvements.

In contrast to quantitative outcomes, qualitative findings more consistently indicated relational rather than symptom‐level change. The thematic synthesis highlighted multi‐family therapy as a nonproblem‐focused and engaging context that fostered intra‐ and inter‐family connection and relational understanding. These changes, including reduced shame, enhanced communication, and recognition of family strengths, may not be adequately captured by global measures of family functioning, offering a plausible explanation for the relatively limited quantitative effects at the family‐system level. This discrepancy may further reflect the slower and context‐dependent nature of systemic change, limited sensitivity of existing measures, and insufficient intervention dose or follow‐up duration.

### 4.2 Cultural Adaptation of Multi‐Family Therapy in Chinese and Singaporean Contexts

Most multi‐family therapy programs in our review were developed through adaptations of Western models to local cultural and service settings, with most adopting the systemic framework proposed by Asen and Scholz (2010). Similar to Western multi‐family therapy, these interventions promote interaction and experience sharing among multiple families to address caregiving challenges and mobilize collective resources. At the same time, Asian multi‐family therapy practices exhibit distinctive features reflecting deeper integration with local cultural norms.

One key area of adaptation concerns the therapist's role. Compared with Western approaches that emphasize therapist decentralization and egalitarian participation, several Asian studies reported more structured arrangements, including pre‐group orientation sessions and clearly defined activity goals (Ma et al. 2017). Therapists often outlined session plans, explained activity rationales, and clarified parents' roles and group objectives (Ma et al. 2022), reflecting cultural expectations of professional authority and facilitating engagement. Importantly, qualitative findings suggest that participants also value a non‐judgemental and supportive therapist stance, which reduces psychological burden and encourages open sharing (Lo and Ma 2024). These positions are not mutually exclusive: therapists may be recognized as authoritative while maintaining a non‐evaluative style. Studies from Singapore further indicate that preferences for authoritative guidance are more commonly expressed by caregivers than by service users (Loh et al. 2021, 2023), possibly reflecting generational differences in views on authority and family hierarchy. Together, rather than drawing a rigid distinction between “Western” and “Asian” approaches, these findings underscore the importance of therapists maintaining a flexible role throughout the intervention process.

Worries about “face” might inhibit self‐disclosure in group‐based multi‐family therapy were not strongly supported. Parents frequently described multi‐family therapy as a psychologically safe space and reported reduced stigma, which increased their willingness to share personal experiences and family difficulties. This suggests that multi‐family therapy may transcend culturally rooted shame‐related barriers and that its effectiveness is relatively robust across cultural contexts.

At the level of intervention activities, Asian multi‐family therapy practices emphasized pragmatism and flexibility. For example, parent–child dyadic mindfulness exercises were sometimes found impractical due to parental exhaustion and children's attentional difficulties, leading practitioners to deliver exercises separately to parents and children (Ma et al. 2018). Other adaptations included nature‐based programmes in high‐density urban settings to alleviate family stress.

### 4.3 Limitations

This review has several limitations. First, the geographical scope of the included studies was limited, with all 17 studies (reported across 18 reports) originating from Hong Kong, the Chinese Mainland, and Singapore. No studies from other East and Southeast Asian countries or regions—such as Japan, South Korea, Indonesia, Thailand, or others—met the inclusion criteria. This geographical concentration limits the generalizability of the findings, and the results should be interpreted as primarily reflecting multi‐family therapy implementation in Chinese cultural contexts. Second, the included studies showed substantial methodological heterogeneity in multi‐family therapy models, intervention duration and structure, cultural adaptations, and outcome measures, which limited cross‐study comparability and warrants caution in interpreting overall effects. Third, to provide a comprehensive overview of multi‐family therapy research in East and Southeast Asia, studies were not excluded based on methodological quality but were instead appraised and retained. Consequently, the conclusions may be influenced by variability in study quality. Fourth, although a review protocol was prepared during the planning stage of the review, the review was not prospectively registered in PROSPERO, OSF, or another review registry. Notably, only three studies employed randomized controlled designs, with most lacking comparison groups. Although pre–post designs offer useful information about potential within‐group change, they do not allow robust inferences about whether multi‐family therapy is superior to other interventions. Finally, several studies delivered multi‐family therapy as an adjunct to concurrent treatments, potentially confounding observed effects and limiting the ability to isolate multi‐family therapy's unique contribution and mechanisms of change (Gelin et al. 2018).

### 4.4 Clinical and Research Implications

This review provides preliminary support for the application of multi‐family therapy in the Chinese Mainland, Hong Kong, and Singapore, suggesting that it can be implemented across a range of service settings and is associated with improvements in individual‐level outcomes. Qualitative evidence further indicates that multi‐family therapy may offer important subjective and process‐level benefits, including reduced shame and stigma, enhanced support and connectedness, and improved communication and relational understanding.

Clinically, it may therefore be more realistic to prioritize short‐term symptom improvement as a primary aim of multi‐family therapy, while conceptualizing family‐level change as a longer‐term process. The relatively weaker quantitative evidence for family‐system outcomes does not necessarily indicate an absence of such change, but may partly reflect limitations in outcome assessment. Several included studies did not systematically assess family‐level outcomes (Loh et al. 2021, 2023; Zhuo et al. 2020), potentially underestimating multi‐family therapy's effects. Future research should more consistently include family‐level measures and, informed by qualitative evidence on key change processes, employ more sensitive indicators to clarify multi‐family therapy's active ingredients and pathways of change. Greater methodological rigor, particularly through additional randomized controlled trials, is also needed.

Evidence regarding the economic benefits of multi‐family therapy remains limited. Future studies should incorporate cost‐effectiveness analyses and compare multi‐family therapy with usual care in terms of healthcare utilization and caregiving‐related costs to better determine its resource implications.

Attention to cultural sensitivity, greater geographical diversity, and increased focus on underrepresented clinical populations are needed to further strengthen the evidence base. Notably, despite the prominence of eating disorder‐focused multi‐family therapy in the Western literature, no such studies were identified in the present review, highlighting an important gap and priority for future research in East and Southeast Asia.

## 5 Conclusion

This review provides the first synthesis of evidence on multifamily therapy across the included East and Southeast Asian settings (Chinese Mainland, Hong Kong, and Singapore). The findings indicate that multi‐family therapy is associated with more consistent improvements in individual‐level outcomes, whereas the evidence for family‐system‐level outcomes remains comparatively weaker. These findings should be interpreted cautiously; stronger evidence for individual outcomes stems from both RCTs and non‐randomized studies, whereas family‐system outcomes were only assessed in non‐randomized and feasibility studies with varied measures and inconsistent results. Readers should consider study designs and methodological quality when interpreting each outcome. Qualitative data indicate that meaningful relational changes are central to participants' experiences, as existing quantitative measures may not fully capture these changes. Overall, the findings provide preliminary support for the feasibility and cultural adaptability of multi‐family therapy in the East and Southeast Asian settings represented in this review, which were limited to the Chinese Mainland, Hong Kong, and Singapore. Broader claims across East and Southeast Asia would require more geographically diverse and methodologically rigorous studies. Variations in treatment delivery across studies reflect context‐sensitive adaptations, underscoring the flexibility of multi‐family therapy in cross‐cultural applications. As such, multi‐family therapy represents not only a symptom‐focused intervention but also a relational process that is embedded within specific cultural contexts.

## Funding

The authors have nothing to report.

## Ethics Statement

The authors have nothing to report.

## Supporting information


Supporting File

## Data Availability

The data that support the findings of this study are available in the supplementary material of this article.
