# Supplementary material for: Multi‐Family Therapy in East and Southeast Asia—A Systematic Review and Meta‐Synthesis
Source: J Marital Fam Ther. 2026 Aug 19;52(4):e70166. doi: 10.1111/jmft.70166 (PMC13487648; doi:10.1111/jmft.70166)
Supplement: Supplementary file 1 [file JMFT-52-0-s001.docm]

**Supplementary Information**

**Search terms:**

 Embase <1974 to 2025 April 21>

1 (multi*family therap* or multi family therap* or MFT or multiple family therap* or multi*family group* or multiple family group* or multiple-family group*).ab,mf,ti. 1533 2 (Asia or China or Hong Kong or Taiwan or Macau or Macao or singapore or malaysia or thailand or vietnam or East Asia or ASEAN or Chinese or Taiwanese or Singaporean or Malaysian or Thai or Vietnamese or japan or japanese or Korea or Korean or burma or myanmar or laos or cambodia or Brunei or Timor-Leste or Philippines or North korea or indonesia*).ab,mf,ti. 1788675 3 1 and 2 84 4 limit 3 to human 59

APA PsycInfo <1806 to April 2025 Week 1>

1 (multi*family therap* or multi family therap* or MFT or multiple family therap* or multi*family group* or multiple family group* or multiple-family group*).ab,mf,ti. 1368 2 (Asia or China or Hong Kong or Taiwan or Macau or Macao or singapore or malaysia or thailand or vietnam or East Asia or ASEAN or Chinese or Taiwanese or Singaporean or Malaysian or Thai or Vietnamese or japan or japanese or Korea or Korean or burma or myanmar or laos or cambodia or Brunei or Timor-Leste or Philippines or North korea or indonesia*).ab,mf,ti. 214504 3 1 and 2 48 4 limit 3 to human 48

Ovid MEDLINE(R) ALL <1946 to April 21, 2025>

1 (multi*family therap* or multi family therap* or MFT or multiple family therap* or multi*family group* or multiple family group* or multiple-family group*).ab,ti. 1328 2 (Asia or China or Hong Kong or Taiwan or Macau or Macao or singapore or malaysia or thailand or vietnam or East Asia or ASEAN or Chinese or Taiwanese or Singaporean or Malaysian or Thai or Vietnamese or japan or japanese or Korea or Korean or burma or myanmar or laos or cambodia or Brunei or Timor-Leste or Philippines or North korea or indonesia*).ab,ti. 1292602 3 1 and 2 72 4 limit 3 to humans 44

Global Health <1973 to 2025 Week 16>

1 (multi*family therap* or multi family therap* or MFT or multiple family therap* or multi*family group* or multiple family group* or multiple-family group*).ab,ti. 172 2 (Asia or China or Hong Kong or Taiwan or Macau or Macao or singapore or malaysia or thailand or vietnam or East Asia or ASEAN or Chinese or Taiwanese or Singaporean or Malaysian or Thai or Vietnamese or japan or japanese or Korea or Korean or burma or myanmar or laos or cambodia or Brunei or Timor-Leste or Philippines or North korea or indonesia*).ab,ti. 554366 3 1 and 2 18

45 duplicates

In the China National Knowledge Infrastructure (CNKI) database, an OR-combined subject search was conducted using the terms “多家庭团体” (multi-family group), “多家庭团体治疗” (multi-family group therapy), “多家庭治疗” (multi-family therapy), and “多家庭小组” (multi-family group), i.e. (Subject: 多家庭团体 OR Subject: 多家庭团体治疗 OR Subject: 多家庭治疗 OR Subject: 多家庭小组), with no limits on publication year, yielding a total of 15 records.

**Table S1. Summary of quality appraisal results**

| **Study Type** | **MMAT Criteria** | **(Chien & Chan, 2004)** | **(Zhuo et al., 2020)** |  |  |  |  |  |
| --- | --- | --- | --- | --- | --- | --- | --- | --- |
| Quantitative-RCT | 2.1. Is randomization appropriately performed? | Yes | CT |  |  |  |  |  |
|  | 2.2. Are the groups comparable at baseline? | Yes | Yes |  |  |  |  |  |
|  | 2.3. Are there complete outcome data? | Yes | Yes |  |  |  |  |  |
|  | 2.4. Are outcome assessors blinded to the intervention provided? | Yes | Yes |  |  |  |  |  |
|  | 2.5 Did the participants adhere to the assigned intervention? | Yes | Yes |  |  |  |  |  |
| **Study Type** | **MMAT Criteria** | **(Lai et al., 2021)** | **(Ma et al., 2018)** | **(Ma et al., 2022)** | **(Ma et al., 2023)** | **(Wang et al., 2016)** | **(Zhang et al., 2025)** | **(Liu et al., 2015)** |
| Quantitative non randomized | 3.1. Are the participants representative of the target population? | No | No | CT | CT | CT | Yes | No |
|  | 3.2. Are measurements appropriate regarding both the outcome and intervention (or exposure)? | Yes | Yes | Yes | Yes | Yes | Yes | Yes |
|  | 3.3. Are there complete outcome data? | CT | No | No | No | Yes | Yes | Yes |
|  | 3.4. Are the confounders accounted for in the design and analysis? | No | Yes | Yes | Yes | CT | CT | Yes |
|  | 3.5. During the study period, is the intervention administered (or exposure occurred) as intended? | Yes | Yes | Yes | Yes | CT | Yes | Yes |
| **Study Type** | **MMAT Criteria** | **(Lo et al., 2024)** | **(Loh et al., 2023)** | **(Ma et al., 2019)** |  |  |  |  |
| Mixed Methods | 5.1. Is there an adequate rationale for using a mixed methods design to address the research question? | Yes | Yes | Yes |  |  |  |  |
|  | 5.2. Are the different components of the study effectively integrated to answer the research question? | Yes | Yes | Yes |  |  |  |  |
|  | 5.3. Are the outputs of the integration of qualitative and quantitative components adequately interpreted? | Yes | CT | Yes |  |  |  |  |
|  | 5.4. Are divergences and inconsistencies between quantitative and qualitative results adequately addressed? | CT | CT | Yes |  |  |  |  |
|  | 5.5. Do the different components of the study adhere to the quality criteria of each tradition of the methods involved? | No | Yes | Yes |  |  |  |  |
| **Study Type** | **MMAT Criteria** | **(Lo & Ma, 2023)** | **(Lo & Ma, 2024)** | **(Loh et al., 2021)** | **(Ma et al., 2011)** | **(Wan et al., 2016)** | **(Wong et al., 2019)** |  |
| Qualitative | 1.1. Is the qualitative approach appropriate to answer the research question? | Yes | Yes | Yes | Yes | Yes | Yes |  |
|  | 1.2. Are the qualitative data collection methods adequate to address the research question? | Yes | Yes | Yes | Yes | Yes | Yes |  |
|  | 1.3. Are the findings adequately derived from the data? | Yes | CT | Yes | CT | Yes | Yes |  |
|  | 1.4. Is the interpretation of results sufficiently substantiated by data? | CT | Yes | CT | Yes | Yes | CT |  |
|  | 1.5. Is there coherence between qualitative data sources, collection, analysis and interpretation? | Yes | Yes | Yes | Yes | Yes | Yes |  |

Note: CT = Can’t tell

**Table S2. Supplementary notes regarding the quality assessment criteria that were rated as ‘no’.**

| **Study (Author, Year)** | **Study Design** | **Quality Assessment criteria** | **Rating** | **Key Comments** |
| --- | --- | --- | --- | --- |
| (Lai et al., 2021) | Quantitative non-randomized | 3.1 | No | Clinic-based convenience sample with self-selection and restrictive eligibility (commitment/Cantonese/stability), limiting representativeness. |
|  |  | 3.4 | No | A large baseline imbalance in child medication use (74.4% vs 21.4%) was not controlled for. |
| (Ma et al., 2018) | Quantitative non-randomized | 3.1 | No | Referral-based, voluntary recruitment (talk attendees) with restrictive eligibility (commitment/Cantonese/stability) limits representativeness. |
|  |  | 3.3 | No | A 21.6% nonresponse rate (missing follow-up data not clearly addressed). |
| (Ma et al., 2022) | Quantitative non-randomized | 3.3 | No | Completion of all three assessments was low (21/51 EG; 6/25 CG) |
| (Ma et al., 2023) | Quantitative non-randomized | 3.3 | No | High attrition/nonresponse (EG: 14.8% at T2; 48.1% at T3; CG: 60.5% at T2; 79.1% at T3) |
| (Lo et al., 2024) | Mixed-methods | 5.5 | No | Qualitative methods were described, but quantitative quality is limited (small sample, single-item measure, no clustering adjustment). |

Table S3. Summary of quantitative outcome measures

| **Author/Year** | **Disorder** | **Outcome measures used** | **References for questionnaires** |
| --- | --- | --- | --- |
| (Chien & Chan, 2004) | Schizophrenia | **family caregivers:**  Specific Level of Functioning Scale (SLOF); Family Support Services Index (FSSI). | **SLOF:** Lee, K. L. (1999). *Study on the effect of the pre-discharge rehabilitation program on the level of functioning of chronic schizophrenic patients in Hong Kong* [Unpublished dissertation]. The Chinese University of Hong Kong. **FSSI:** Heller, T., & Factor, A. (1991). Permanency planning for adults with mental retardation living with family caregivers. *American Journal of Mental Retardation: AJMR*, *96*(2), 163–176. |
| (Zhuo et al., 2020) | Schizophrenia (stable phase) | **patients:** Life Satisfaction Index A (LSIA); Self-Rating Depression Scale (SDS); Self-Rating Anxiety Scale (SAS ).  **researchers:**  Personal and Social Performance Scale (PSP). | **PSP:** Morosini, P. L., Magliano, L., Brambilla, L., Ugolini, S., & Pioli, R. (2000). Development, reliability and acceptability of a new version of the DSM-IV Social and Occupational Functioning Assessment Scale (SOFAS) to assess routine social functioning. *Acta psychiatrica Scandinavica*, *101*(4), 323–329.  Qiao, Y., He, Y.-L., Zhao, J.-P., et al. (2012). Reliability and validity of the Personal and Social Performance Scale. *China Medical Herald*, 9(26), 11–14. |
| (Lai et al., 2021) | ADHD | **parents:** Strengths and Weaknesses of ADHD Symptoms and Normal Behavior Scale (SWAN); Parent–Child Relationship Scale (PCR); Parenthood Stress Questionnaire (PSQ); Parenting Sense of Competence Scale (PSOC). | **SWAN:** Swanson, J., Schuck, S., Mann, M., Carlson, C., Hartman, K., Sergeant, J., … Wasdell, M. (2005). *Categorical and dimensional definitions and evaluations of symptoms of ADHD: The SNAP and the SWAN rating scales*.  **PCR:** Fine, M. A., Moreland, J. R., & Schwebel, A. I. (1983). Long-term effects of divorce on parent–child relationships. *Developmental Psychology, 19*, 703–713.  **PSOC:** Gibaud-Wallston, J., & Wandersmann, L. P. (1978). *Development*  *and utility of the Parenting Sense of Competence Scale*. Washington, DC: John F. Kennedy Center for Research on  Education and Human Development.  **PSQ:**Ostberg, M., & Hagekull, B. (2000). A structural modeling  approach to the understanding of parenting stress. *Journal of*  *Clinical Psychology*, *29*, 615-625. |
| (Liu et al., 2015) | Internet addiction | **patients:**  Adolescent Pathological Internet Use Scale (APIUS); Chinese version of Closeness to Parents Scale(CPS); chinese version of the Parent–Child Communication Scale (PCCS); College Students' Psychological Needs and Fulfillment Scale(CSPNFS). | **APIUS:** Lei, L., & Yang, Y. (2007). The development and validation of adolescent pathological Internet use scale. *Acta Psychologica Sinica*, *39*, 688–696.  **CPS:** Liu, Q.-X., Fang, X.-Y., Zhou, Z.-K., Zhang, J.-T., & Deng, L.-Y. (2013). Perceived Parent-Adolescent Relationship, Perceived Parental Online Behaviors and Pathological Internet Use among Adolescents: Gender-Specific Differences. *PLOS ONE*, *8*(9), e75642.  **PCCS:** Liu, Q.-X., Fang, X.-Y., Deng, L.-Y., & Zhang, J.-T. (2012). Parent–adolescent communication, parental Internet use and Internet-specific norms and pathological Internet use among Chinese adolescents. *Computers in Human Behavior*, *28*(4), 1269–1275.  **CSPNFS:** Jingjing, W., Zhang, J., Liu, Q.-X., Deng, L., & Fang, X. (2010). Development of college students’ psychological need internet gratification questionnaire. *Studies of Psychology and Behavior*, *8*, 118–125. |
| (Ma et al., 2018) | ADHD | **parents:** SWAN consists of two subscales: AHDH-Hyperactivity/Impulsivity  (ADHD-HI) and ADHD-Inattentive (ADHD-I); PCR; PSOC; Parenting Stress Index (PSI); Stoner Hope Scale (PSH);  Perceived Social Support Scale (PSS). | **SWAN:** Swanson, J., Mann-Porter, M., Carlson, C., Hartman, C., & Sergeant, J. (2004). Categorical and dimensional definitions and evaluations of symptoms of ADHD: History of the SNAP and SWAN rating scales. *The International Journal of Educational and Psychological Assessment*, *10*, 51–70. **PSI:** Ostberg, M., & Hagekull, B. (2000). A structural modeling approach to the understanding of parenting stress.  *Journal of Clinical Child Psychology*, *29*(4), 615–625.  **PSI:** Ma, J. L. C., Wong, T. K. Y., Lau, Y. K., & Lai, L. Y. (2011). Parenting stress and perceived family functioning of Chinese parents in Hong Kong: Implications for social work practice. *Asian Social Work and Policy Review*, *5*,(3), 160–180.  **PSH:** Farran, C. J., Salloway, J. C., Clark, D. C., Horn, B. J., Hayes, E. R., & Copstead, E. (1990). Measurement of hope  in a community-based older population. *Western Journal of Nursing*, *12*(1), 42–59.  **PSS:** Ma, J. L. C. (1996). Desired and perceived social support from family, friends, and health professionals: A panel  study in Hong Kong patients with nasopharyngeal carcinoma. *Journal of Psychosocial Oncology*, *14*(3), 47–68. |
| (Ma et al., 2022) | Depression | **patients:** Ohio Youth Problems and Functioning Scales (OYPFS);  Perceived Competence Scale (PCS); Student Social Support Scale (SSSS); PCR(Fine et al., 1983). | **OYPFS:** Ogles, B. M., Melendez, G., Davis, D. C., & Lunnen, K. M. (2001). The Ohio scales: Practical outcome assessment.  *Journal of Child and Family Studies*, *10*(2), 199–212.  **PCS:** Harter, S. (1982). The perceived competence scale for children. *Child Development*, *53*(1), 87–97.  **SSSS:** Demaray, M. K., & Elliott, S. N. (2001). Perceived social support by children with characteristics of Attention  Deficit/Hyperactivity Disorder. *School Psychology Quarterly*, *16*(1), 68–90.  **PCR:** Fine, M. A., Moreland, J. R., & Schwebel, A. I. (1983). Long-term effects of divorce on parent–child relationships. *Developmental Psychology, 19*, 703–713. |
| (Ma et al., 2023) | Depression | **Parents:** The Brief Symptom Inventory (BSI); PCR (Fine et al., 1983);  PSOC(Gibaud-Wallston & Wandersman, 1978) PSI(Ostberg & Hagekull, 2000);  General Family Functioning Scale (GFFS); PSS(Ma, 1996). | **GFFS:** Epstein, N. B., Baldwin, L. M. and Bishop, D. S. (1983) The McMaster family assessment device. *Journal of Marital*  *and Family Therapy*, 9(2), 171–80  **BSI:** Derogatis, L. R. (2000) *Brief Symptom Invetory-18 (BSI-18): Administration, Scoring, and procedures manual* Minneapolis, MN: National Computer systems. |
| (Wang et al., 2016) | Childhood schizophrenia remission phase | **patients:** Positive and Negative Syndrome Scale (PANSS); **researchers:**  Personal and Social Performance Scale(PSP). | **PANSS:** Si, T., Yang, J., Shu, L., et al. (2004). Reliability and validity of the Chinese version of the Positive and Negative Syndrome Scale (PANSS). *Chinese Mental Health Journal*, *18*(1), 45–47.  **PSP:** Si, T., Shu, L., Tian, C., et al. (2009). Reliability and validity of the Chinese version of the Personal and Social Performance Scale in patients with schizophrenia. *Chinese Mental Health Journal*, *23*(11), 790–794. |
| (Zhang et al., 2025) | Bipolar disorder (BD), remission phase | **patients:**  Morisky Medication Adherence Scale(MMAS);  36-Item Short From Health Survey(SF-36).  **Completed by researcher:**  Personal and Social Performance Scale(PSP). | **PSP:** Qiao, Y., He, Y., Zhao, J., et al. (2012). Reliability and validity of the Personal and Social Performance Scale. *China Medical Herald*, *9*(26), 11–14. **MMAS:**Yang, T., Tong, Y., Yin, X., et al. (2016). An evaluation of the application of the Morisky Medication Adherence Scale and the Family APGAR questionnaire in patients with tuberculosis. *Chinese Journal of Social Medicine*, *33*(6), 580–582. **SF-36:** Wang, K. (2012). The Short Form Health Survey (SF-36). *Chinese Journal of Neurosurgery*, *28*(6), 571. |
| (Lo et al., 2024) | ASD | **parents and patients:** Self-administered client satisfaction questionnaire (CSQ). | CSQ includes Treatment engagement, Cross-family therapeutic alliance. |
| (Loh et al., 2023) | First-Episode Psychosis (FEP) | **parents and patients:**  Self-developed Questionnaire 1 **EPIP team members:**  Self-developed Questionnaire 2 | Questionnaire 1 (19 Likert-scale items + 3 open-ended) on structure, content, and effects of MFT; Questionnaire 2 (4 open-ended items) on understanding of MFT and recruitment barriers. |
| (Ma et al., 2019) | ADHD | **patients:** Children’s Hope Scale (CHS); PCS (Harter, 1982);  PCR(Fine et al., 1983); SSSS(Demaray and Elliot, 2001). | **CHS:** Snyder, C. R., Hoza, B., Pelham, W. E., Rapoff, M., Ware, L., Danovsky, M.,  Highberger, L., Ribinstein, H. and Stahl, K. J. (1997) The development and  validation of the Children’s Hope Scale. *Journal of Pediatric Psychology*, *22*(3):  399–421. |
|  |  |  |  |

Table S4. Summary of quantitative outcomes

| Author/Year | Disorder | Outcome measures used | Effectiveness |
| --- | --- | --- | --- |
| (Chien & Chan, 2004) | Schizophrenia | family caregivers:  Specific Level of Functioning Scale (SLOF); Family Support Services Index (FSSI). | By the 12-month follow-up, MFT group showed greater improvements in SLOF;larger reductions in rehospitalization than other groups;between-group differences over time were significant (MANOVA: *F* = 4.89, *p* = .005, *ηp*² = .18). |
| (Zhuo et al., 2020) | Schizophrenia (stable phase) | patients: Life Satisfaction Index A (LSIA); Self-Rating Depression Scale (SDS); Self-Rating Anxiety Scale (SAS ).  researchers:  Personal and Social Performance Scale (PSP). | At post-intervention, MFT group showed significant improvements in PSP (*F* = 11.94, *p* = .034) and LSIA (*F* = 6.58, *p* = .009), and reduction in SAS (*F* = 15.10, *p* < .001). |
| (Lai et al., 2021) | ADHD | parents: Strengths and Weaknesses of ADHD Symptoms and Normal Behavior Scale (SWAN); Parent–Child Relationship Scale (PCR); Parenthood Stress Questionnaire (PSQ); Parenting Sense of Competence Scale (PSOC). | At post-intervention, MFT group reported a significant reduction in SWAN scores (*p* < .05). No significant differences were observed in PSQ and PSOC for either group. Fathers’ PCR increased in both the MFT group (*p* < .05, *d* = 0.42) and the control group (*p* < .05, *d* = 0.67). |
| (Liu et al., 2015) | Internet addiction | patients:  Adolescent Pathological Internet Use Scale (APIUS); Chinese version of Closeness to Parents Scale(CPS); chinese version of the Parent–Child Communication Scale (PCCS); College Students' Psychological Needs and Fulfillment Scale(CSPNFS). | At post-intervention, MFT group showed a significant reduction in APIUS scores (*t* = −7.79, *p* < .001) and in CSPNFS scores (t = −2.57, *p* < .05),；significant improvements in CPS (*t* = 2.89, *p* < .01) and PCCS (*t* = 4.08, *p* < .001) compared with the control group. The effects remained significant at the 3-month follow-up. |
| (Ma et al., 2018) | ADHD | parents: SWAN consists of two subscales: AHDH-Hyperactivity/Impulsivity  (ADHD-HI) and ADHD-Inattentive (ADHD-I); PCR; PSOC; Parenting Stress Index (PSI); Stoner Hope Scale (PHS);  Perceived Social Support Scale (PSS). | At post-intervention, MFT group reported a significant reduction in SWAN scores (*t*=5.07, *p* < .001); MFT showed a significant positive effect on parents' perception of children's ADHD symptoms, with moderate effect sizes (*d* = .42–.57), all *p* < .001. No significant improvements in PCR(*d* = .10), PSI(*d* = .18), PSOC(*d* = .16), PHS(*d* = .44) and PSS(*d* = .32). |
| (Ma et al., 2022) | Depression | patients: Ohio Youth Problems and Functioning Scales (OYPFS);  Perceived Competence Scale (PCS); Student Social Support Scale (SSSS); PCR(Fine et al., 1983). | At post-intervention and 3-month follow-up, no significant between-group difference was found for SSSS(*t*=1.63, *p*=.107); and Youth problem severity (*t*=-2.33, *p*=.022) |
| (Ma et al., 2023) | Depression | Parents: The Brief Symptom Inventory (BSI); PCR (Fine et al., 1983);  PSOC(Gibaud-Wallston & Wandersman, 1978) PSI(Ostberg & Hagekull, 2000);  General Family Functioning Scale (GFFS); PSS(Ma, 1996). | At post-intervention, the MFT group showed a greater reduction in BSI (*d* = 0.38), and this effect remained at the 3-month follow-up;  No significant differences in PCR (*d* = .09), PSI(*d* = .12), or GFFS(*d* = .02) between MFT and control group. |
| (Wang et al., 2016) | Childhood schizophrenia remission phase | patients:  Positive and Negative Syndrome Scale (PANSS); researchers:  Personal and Social Performance Scale(PSP). | At post-intervention, MFT group showed significantly lower PANSS scores (*F*=25.6, *p* <0.01) and higher PSP scores (*F*=30.4, *p* <0.01) than other groups;this effect remained at the 3-month follow-up. |
| (Zhang et al., 2025) | Bipolar disorder (BD), remission phase | patients:  Morisky Medication Adherence Scale(MMAS);  36-Item Short From Health Survey(SF-36).  Completed by researcher:  Personal and Social Performance Scale(PSP). | At 2-month follow-up, MFT group showed significant improvement in PSP (*t*=5.532, *p* <0.05); and SF-36 (*p* <0.05);  Higher medication adherence (90.24% vs 69.05%, *χ2*=5.72, *p* <0.05). |
| (Lo et al., 2024) | ASD | parents and patients: Self-administered client satisfaction questionnaire (CSQ). | At post-intervention, cross-family therapeutic alliance (*r*=.69, *p*=.001) and treatment engagement (*r*=.59, *p*=.01) positively correlated with the enhanced family relationships |
| (Loh et al., 2023) | First-Episode Psychosis (FEP) | parents and patients:  Self-developed Questionnaire 1 EPIP team members:  Self-developed Questionnaire 2 | At post-intervention, perceived MFT program effects (*M* = 3.66, *SD* = 0.57) and perceived MFT program usefulness of contents were high(*M* = 3.17, *SD* = 0.42). |
| (Ma et al., 2019) | ADHD | patients: Children’s Hope Scale (CHS); PCS (Harter, 1982);  PCR(Fine et al., 1983); SSSS(Demaray and Elliot, 2001). | At post-intervention, no significant between-group difference was found for PCS(*F*=1.11, *d*=.21); PCR(*F*=.45, *d*=.14); SSSS frequency(*F*=.06, *d*=.06) |

Table S5. Qualitative quotes from the included studies.

| **Study** | **Title** | **Number of themes** | **Theme** |
| --- | --- | --- | --- |
| **(Ma et al., 2019)；ADHD；Hong Kong** | Multiple family therapy for Chinese families of children with attention deficit hyperactivity disorder  (ADHD): treatment efficacy from the children’s perspective and their subjective experiences | 5 | Fun  Building friendships through common experiences  Happy family time  Safe space  Positive parental responses and communications |
| **(Lo et al., 2024) Autism Spectrum Disorder**  **Hong Kong** | The Feasibility and the Therapeutic Process Factors of Online vs. Face-to-Face Multifamily Therapy for Adults with High-Functioning Autism  Spectrum Disorder in Hong Kong: A Multi-Method Study | 2 | Cross-Family Interactional Context  Subtheme: Observational Learning  Authentic Engagement with People with HF-ASD  Subthemes:  Direct Exchange  Psychological Safety |
|  |  |  |  |
|  |  |  |  |
|  |  |  |  |
|  |  |  |  |
|  |  |  |  |
|  |  |  |  |
|  |  |  |  |
|  |  |  |  |
| **(Loh et al., 2021) First-Episode Psychosis (FEP) Singapore** | Development of Multi-Family Therapy for first episode psychosis in Singapore | 3 | Support and learning through sharing of experiences    Positive experience with MFT    Suggestions for improvement.  Subthemes:  (1) including more interactive and fun games, (2) incorporating success stories of clients and carers, (3) having more time for separate group discussions and (4) reducing the discussion time in the multiple family context |
|  |  |  |  |
|  |  |  |  |
|  |  |  |  |
|  |  |  |  |
| **(Lo & Ma, 2022) ADHD Hong Kong** | Use of nature in a multi-family therapy  for Chinese families of adolescents with  attention deficit hyperactivity disorder:  A photo-elicitation study | 6 | A change in the group environment  Subthemes:   - Change from urban space to countryside - Temporary escape from daily routine tasks - Living together with other families   Spaciousness  Subthemes:  Living together with other families  A large piece of grassland  A wide sea view  Darkness  Subthemes:  Family solo walk  Feeling of being unsafe      An increase in the self-efficacy  of the adolescents with  ADHD  Subthemes:  Overcoming challenging tasks and frustration  Receiving support from parents    An increase in emotional control of the adolescents with ADHD  Subthemes:  Private space  Psychological autonomy  Strengthening the sense of family agency of the parents  Subthemes:  Overcoming challenging tasks with family members  Awareness of family strengths |
| **(Lo & Ma, 2023) ADHD Hong Kong** | Generating mutual support in multifamily therapy to  promote father involvement and family communication  quality of Chinese families of adolescents with  Attention Deficit Hyperactivity Disorder:  A qualitative study | 3 | Low level of hierarchy in the therapist–client relationship      Increased acceptance in the families      Increased father involvement and open communication in the families |
| **(Loh et al., 2021) First episode psychosis Singapore** | Multi-Family Therapy for First Episode Psychosis: Experiences of Families in Singapore | 4 | Therapeutic Processes of MFT      Positive Changes in Family Relationship    Improvements in Coping With Psychosis    Suggestions for Improvements |
|  |  |  |  |
|  |  |  |  |
|  |  |  |  |
|  |  |  |  |
|  |  |  |  |
|  |  |  |  |
|  |  |  |  |
|  |  |  |  |
| **(Ma et al., 2011) Child behavioral/emotional issues: Hong Kong** | An Alternative Way of Helping Chinese Parents With Parenting Difficulties: Learning From the Participants' Feedback on the Multiple Family Groups in Hong Kong | 2 | Perceived Changes at Intrafamilial Level  Subtheme: Change of perception of children’s problems      Perceived Changes at the Interfamilial Level  Subtheme:  Change of parenting attitude and methods  Change of family atmosphere  Expansion of social support  Learning from one another  Surrogate parenting |
|  |  |  |  |
|  |  |  |  |
|  |  |  |  |
|  |  |  |  |
|  |  |  |  |
|  |  |  |  |
|  |  |  |  |
|  |  |  |  |
|  |  |  |  |
|  |  |  |  |
|  |  |  |  |
| **(Wan et al., 2016) ADHD Hong Kong** | The Subjective Experiences of Attention-Deficit/ Hyperactivity Disorder of Chinese Families in  Hong Kong: Co-Construction of Meanings in  Multiple Family Groups | 5 | Parents’ Feelings of Ambivalence about  Medication  ADHD Medication as a Problem-Solving  Strategy    Children’s Will to Behave    “The Medicated Self Is Part of Me”  Children’s Moral Awareness |
|  |  |  |  |
|  |  |  |  |
|  |  |  |  |
|  |  |  |  |
|  |  |  |  |
|  |  |  |  |
| **(Wong et al., 2019) parent-child relationship problems Hong Kong** | The impact of poverty on children in out-of-home care services in a Chinese context and the application of multiple family group therapy to enrich their family lives | 2 | group outcomes  Subtheme:  Change in family relationships;  Building a support network  Feedback on group design and implementation |
| **(Ma et al., 2020) High-functioning autism (HFA) Hong Kong** | Repairing the Parent–Child Relationship for a Hong Kong Chinese  Family of an Adult Daughter with High Functioning Autism (HFA)  Through Structural Family Therapy and Multiple Family Therapy | 2 | Treatment Outcome of Family Therapy: Occasional  Presence in the Sitting Room  Family Perspective: The Therapeutic Effects of SFT  and MFT on the Parent–Child Relationships |

## **Supplementary information:**

## **Multifamily Therapy in East and Southeast Asia – a review and meta-synthesis: Protocol**

***RQ1.*** What are the characteristics of the MFT in East and Southeast Asia including the presenting conditions, settings and cultural adaptations?

***RQ2.*** What are the mental health and family functioning outcomes of these interventions?

***RQ3.*** What are the experiences of individuals and families who participated in MFT?

## **PICOs**

| Population | Intervention | Comparison | Outcome |
| --- | --- | --- | --- |
| Individuals with mental health or neurodevelopmental conditions and their families | Multifamily therapy | Usual care  Control  No comparison | Mental health symptoms  Family relationships |

**Inclusion criteria**

- Peer-reviewed empirical studies (quantitative, qualitative, or mixed-methods)
- Studies on multi-family therapy
- Conducted in East or Southeast Asian countries or regions
- Articles in English or Chinese

**Exclusion criteria**

- Dissertation, conference abstracts, book chapters, study protocols, case reports without evaluative outcome data

**Quality appraisal tool**

- Mixed-method appraisal tool (MMAT)

**Search terms:**

Embase

1 (multi*family therap* or multi family therap* or MFT or multiple family therap* or multi*family group* or multiple family group* or multiple-family group*).ab,mf,ti.

2 (Asia or China or Hong Kong or Taiwan or Macau or Macao or singapore or malaysia or thailand or vietnam or East Asia or ASEAN or Chinese or Taiwanese or Singaporean or Malaysian or Thai or Vietnamese or japan or japanese or Korea or Korean or burma or myanmar or laos or cambodia or Brunei or Timor-Leste or Philippines or North korea or indonesia*).ab,mf,ti.

3 1 and 2

4 limit 3 to human

APA PsycInfo

1 (multi*family therap* or multi family therap* or MFT or multiple family therap* or multi*family group* or multiple family group* or multiple-family group*).ab,mf,ti.

2 (Asia or China or Hong Kong or Taiwan or Macau or Macao or singapore or malaysia or thailand or vietnam or East Asia or ASEAN or Chinese or Taiwanese or Singaporean or Malaysian or Thai or Vietnamese or japan or japanese or Korea or Korean or burma or myanmar or laos or cambodia or Brunei or Timor-Leste or Philippines or North korea or indonesia*).ab,mf,ti.

3 1 and 2

4 limit 3 to human

Ovid MEDLINE(R)

1 (multi*family therap* or multi family therap* or MFT or multiple family therap* or multi*family group* or multiple family group* or multiple-family group*).ab,ti.

2 (Asia or China or Hong Kong or Taiwan or Macau or Macao or singapore or malaysia or thailand or vietnam or East Asia or ASEAN or Chinese or Taiwanese or Singaporean or Malaysian or Thai or Vietnamese or japan or japanese or Korea or Korean or burma or myanmar or laos or cambodia or Brunei or Timor-Leste or Philippines or North korea or indonesia*).ab,ti.

3 1 and 2

4 limit 3 to humans

Global Health

1 (multi*family therap* or multi family therap* or MFT or multiple family therap* or multi*family group* or multiple family group* or multiple-family group*).ab,ti.

2 (Asia or China or Hong Kong or Taiwan or Macau or Macao or singapore or malaysia or thailand or vietnam or East Asia or ASEAN or Chinese or Taiwanese or Singaporean or Malaysian or Thai or Vietnamese or japan or japanese or Korea or Korean or burma or myanmar or laos or cambodia or Brunei or Timor-Leste or Philippines or North korea or indonesia*).ab,ti.

3 1 and 2

**Data extraction**

1. Type of study
2. Disorder
3. Region
4. Setting (inpatient/ outpatient; adult/ children)
5. Cultural sensitivity/ component/ adaptation
6. Approach/ Theoretical framework
7. Design of the MFT group
8. Outcome measures used (validated)
9. Effectiveness/ experiences (qualitative and quantitative)
